# State Transition Modeling in Ultimate Frisbee: Adaptation of a Promising Method for Performance Analysis in Invasion Sports

**DOI:** 10.3389/fpsyg.2021.664511

**Published:** 2021-05-25

**Authors:** Hilary Lam, Otto Kolbinger, Martin Lames, Tiago Guedes Russomanno

**Affiliations:** ^1^Chair of Performance Analysis and Sports Informatics, Department of Sport and Health Sciences, Technical University of Munich, Munich, Germany; ^2^Laboratory for Teaching Computer Science Applied to Physical Education and Sport, Faculty of Physical Education, University of Brasilia, Brasília, Brazil

**Keywords:** Ultimate, Ultimate Frisbee, disc sports, performance analysis, performance indicators, state transition modeling

## Abstract

Although the body of literature in sport science is growing rapidly, certain sports have yet to benefit from this increased interest by the scientific community. One such sport is Ultimate Frisbee, officially known as Ultimate. Thus, the goal of this study was to describe the nature of the sport by identifying differences between winning and losing teams in elite-level competition. To do so, a customized observational system and a state transition model were developed and applied to 14 games from the 2017 American Ultimate Disc League season. The results reveal that, on average, 262.2 passes were completed by a team per game and 5.5 passes per possession. More than two-thirds of these passes were played from the mid zone (39.4 ± 6.57%) and the rear zone (35.2 ± 5.09%), nearest the team’s own end zone. Winning and losing teams do not differ in these general patterns, but winning teams played significantly fewer backward passes from the front zone to the mid zone, nearest the opponent’s end zone than losing teams (mean difference of −4.73%, *t*_(13)_ = −4.980, *p* < 0.001, *d* = −1.16). Furthermore, losing teams scored fewer points when they started on defense, called breakpoints (mean difference of −5.57, *t*_(13)_ = −6.365, *p* < 0.001, *d* = 2.30), and committed significantly more turnovers per game (mean difference of 5.64, *t*_(13)_ = 5.85, *p* < 0.001, *d* = −1.18). Overall, this study provides the first empirical description of Ultimate and identifies relevant performance indicators to discriminate between winning and losing teams. We hope this article sheds light on the unique, but so far overlooked sport of Ultimate, and offers performance analysts the basis for future studies using state transition modeling in Ultimate as well as other invasion sports.

## Introduction

Despite the growing interest in sport science, as seen by the increasing number of articles and journals on the topic over the past couple of decades (Lago, 2009), some lesser-known sports have been neglected. This could be due in part to the pressure felt by some researchers to publish only in top-tier journals where a sport’s popularity could promise greater acclaim. One such sport is Ultimate Frisbee, officially known as Ultimate, a fast-paced, limited contact invasion game played in teams and with a flying disc made of plastic. In a keyword search of “ultimate frisbee” on PubMed and Web of Science, the search results show that less than 20 articles have been published between 2005 and 2020. This is rather surprising, given the sport is played in 85 countries worldwide (World Flying Disc Federation, 2019) and has more than 2,000,000 participants in the United States alone (Sports and Fitness Industry Association, 2020).

So far, the few scientific articles about Ultimate have not focused on performance analysis. Thornton (2004) was one of the first to examine Ultimate as a “lifestyle” sport by exploring the sporting values that differentiate it from other more traditional sports. Subsequently, several more studies were published on the sociocultural aspects of the sport, including the sporting landscape (Griggs, 2009), player norms and practices (Robbins, 2004), and even sport-related drinking behavior (Crocket, 2016). In addition, despite some studies on the physical (Krustrup and Mohr, 2015; Madueno et al., 2017; Palmer et al., 2020) and physiological (Scanlan et al., 2015) demands of the sport, little attention has been given to performance analysis, yet its importance in sports is well-established (O’Donoghue, 2010; Maslovat and Franks, 2019).

Although biomechanical indicators are equally relevant for performance analysis in game sports, the interaction between two opposing teams requires the consideration of other indicators (Lames, 2006; Lames and McGarry, 2007; Garganta, 2009; Korte and Lames, 2018). This interactive behavior is described as a dynamic process, as the actions of either party are subject to change at any time during the match (Lames and McGarry, 2007). Thus, to understand and explain the specific behaviors in game sports, performance analysts must apply techniques that are able to represent or simulate this interaction. One such technique is state transition modeling. A state transition model is a level of modeling that requires not only some input, such as historical performance data, but also some knowledge of the sport’s internal functioning to generate some output, such as future performance (Lames, 2020). This internal functioning is described by a sequence of states, which can be further categorized as starting states, transient states, and absorbing states (Wenninger and Lames, 2016). More importantly, these states must reflect a strictly defined game characteristic, because it is the transitions between these states that provide information about tactical behaviors (Pfeiffer et al., 2010). For example, Pfeiffer et al. (2010) applied state transition modeling to the tactical analysis of stroke techniques in table tennis and found that different strokes contribute to scoring rate, not through winners but by forcing more errors from the opponent. Wenninger and Lames (2016) present an example from table tennis where they derived scoring probabilities as a function of rally length using a customized state transition model. Although there have been reports of such models in invasion games (Hirotsu and Wright, 2003; Forbes and Clarke, 2006), where researchers were able to identify sufficient and appropriate states, none have attempted to apply the model as a spatial representation of the playing field.

The aim of this article is thus twofold. This will be the first study to provide empirical insights into the nature of the sport of Ultimate. In more detail, this study will investigate passing patterns and the spatial structure of possessions. To this end, our second aim was to adapt the existing method of state transition modeling to better suit Ultimate and other invasion sports. We sought to demonstrate the merit of including proper spatial representation of the playing field into state transition models for such sports. Overall, we hope this study will generate further interest in Ultimate within the sports science community, as this sport has been neglected so far.

## Materials and Methods

### Data Collection

#### Sample

The sample entities consisted of 14 games from the 2017 American Ultimate Disc League (AUDL) season (Table 1). The AUDL is a men’s semiprofessional Ultimate league and is only one of several elite-level competitions based out of North America. The sport is also played at the international level and is governed by the World Flying Disc Federation. Ten different teams across the four divisions (East, West, Midwest, and South) and games from both the regular season (weeks 1–17) and the postseason (Championship Weekend) were included. Regular season games were played in various cities across the United States and Canada, but all games from the 2017 Championship Weekend were hosted in Montreal, Canada (home and away team assignments in Table 1 are arbitrary in the postseason). All post-game analyses were performed by a qualified investigator with more than 10 years of elite-level Ultimate experience, using video footage of the games which was made publicly available by AUDL and accessed from YouTube (American Ultimate Disc League, 2017).

**TABLE 1:** Sample entities from the 2017 AUDL season.

**Week**	**Division**	**Home team**	**Away team**
1	West	San Francisco FlameThrowers	San Jose Spiders
2	East	New York Empire	Toronto Rush
2	West	San Jose Spiders	San Francisco FlameThrowers
2	East	DC Breeze	Toronto Rush
4	West	San Francisco FlameThrowers	San Diego Growlers
7	West	San Francisco FlameThrowers	Seattle Cascades
8	West	San Francisco FlameThrowers	Vancouver Riptide
11	East	DC Breeze	Toronto Rush
16	West	San Francisco FlameThrowers	Seattle Cascades
17	East	Toronto Rush	New York Empire
Division Finals	East	Toronto Rush	DC Breeze
Semifinals	West/Midwest	San Francisco FlameThrowers	Madison Radicals
Semifinals	East/South	Toronto Rush	Dallas Roughnecks
Championship	East/West	Toronto Rush	San Francisco FlameThrowers

#### Observational System

To investigate the nature of Ultimate, we chose a multimethod approach combining a series of complementary methods (Anguera et al., 2018). An observational system was designed to systematically annotate the states and state transitions from the state transition model to further analyze performance indicators in Ultimate. The observational system was created in Microsoft Excel (v16.16.23), and the same system was used for all 14 games. Each datasheet contained game identification information, timestamps for every pull, and the state transition model annotations for every possession during the game. These annotations include spatial information about each pass and the result of each possession. In case readers are unfamiliar with Ultimate, the remainder of this paragraph gives a brief introduction to the structure and rules of the sport. The objective of the game is similar to other invasion sports—to score more points than the opposing team while conceding fewer by passing the disc between teammates until it is caught inside the opponent’s end zone. Players are not permitted to run with the disc and can only hold the disc for a limited number of seconds called “stalls.” In the AUDL, players can hold the disc for seven stalls (American Ultimate Disc League, 2019). Each point begins with seven players from each team lined up horizontally in front of their respective end zones. The game is divided into two halves; for the first point of each half, the teams are assigned to either offense or defense. In the AUDL, gameplay is further subdivided into quarters lasting 12 min each (American Ultimate Disc League, 2019). For all subsequent points, the team that scored the previous point starts the next point on defense. The team that starts on defense initiates the point by throwing the disc across the field to the team on offense; this is known as a “pull.” Given that a team always starts on defense after scoring a point, in theory, the rules of Ultimate allow each team equal opportunity to score, as the team that was scored on will start the next point with possession of the disc. As a result, the offensive team’s goal is to maintain possession of the disc and score a point. It is the defensive team’s goal to steal possession of the disc by forcing a turnover with an interception or by an error of the opponent.

### Data Processing

#### General Performance Indicators

From the observational system, nine performance indicators were identified (Table 2). These performance indicators address general match indicators, as well as technical and tactical behaviors in Ultimate. The general match indicators include points scored per game (PPG), breakpoints scored per game (BPG), and possession opportunity (PO%). Breakpoints are points that were scored by the team who pulled the disc. PO% refers to the number of times a team receives the pull and starts with possession of the disc. The technical indicators include total turnovers per game (TTPG), unforced turnovers per game (UTPG), and forced turnovers per game (FTPG). The tactical indicators include completed passes per game (CPG), average number of passes per possession (PPP), and turnover-to-point conversion efficiency (TTPCE%). TTPCE% represents a team’s ability to score during the subsequent possession after a turnover is committed by the opponent.

**TABLE 2:** Identified performance indicators.

Points scored per game (PPG)	A point is scored when a player on the offensive team catches the disc inside the opponent’s end zone
Break points scored per game (BPG)	A break point refers to a point scored by the team who pulled the disc (i.e., started the point on defense)
Completed passes per game (CPG)	A completed pass is a successful exchange of disc possession between players of the same team. A successful exchange refers to full control of the disc (i.e., no fumbling)
Average number of passes per possession (PPP)	A possession is an uninterrupted period where a team maintains control of the disc through a sequence of completed passes
Total turnovers per game (TPG)	A turnover is a loss of disc possession.
Unforced turnovers per game (UTPG)	An unforced turnover is the loss of disc possession due to an error (e.g., disc is dropped, disc is thrown out of bounds)
Forced turnovers per game (FTPG)	A forced turnover is the loss of disc possession due to active defensive effort (e.g., interception, double team)
Turnover-to-point conversion efficiency (TTPCE%)	The turnover-to-point conversion efficiency is a team’s ability to score immediately after a turnover by the opponent. The number of points scored after a turnover is divided by the total number of turnovers by the opponent, expressed as a percentage
Possession opportunity (PO%)	The possession opportunity is the number of times a team starts with the disc (i.e., the opponent pulls) divided by the total number of possessions in the game, expressed as a percentage

#### State Transition Modeling

**FIGURE 1:**
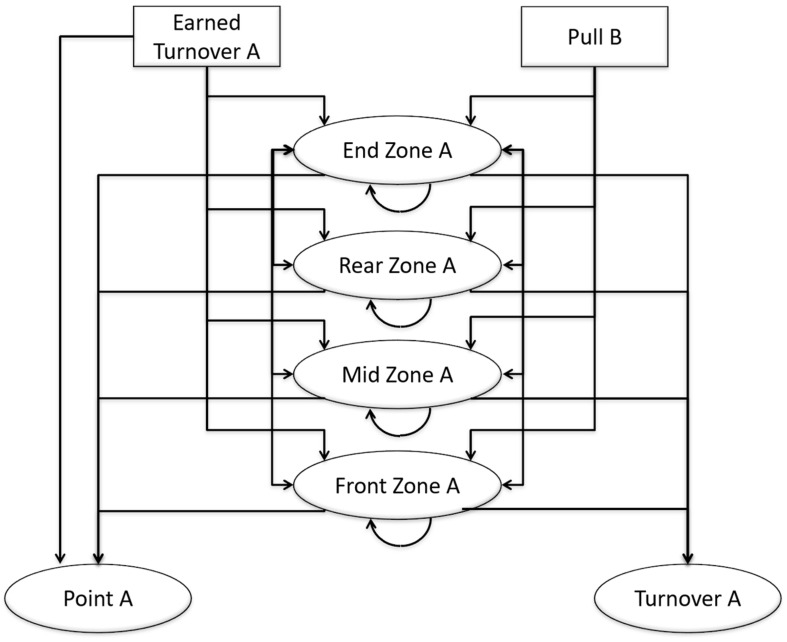
State transition model for analysis of tactical behavior in Ultimate. Labels correspond to initial disc possession by Team A. The same model can be used for initial disc possession by Team B by switching the labels from B→A, A→B.

**FIGURE 2:**
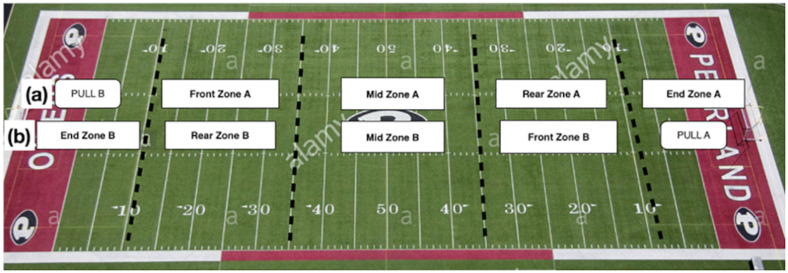
Transient states in the state transition model for Ultimate. **(a)** When Team B pulls and Team A starts with possession of the disc. **(b)** When Team A pulls and Team B starts with the disc.

The concept of state transition modeling was adapted for the analysis of tactical passing behavior in Ultimate. This state transition model, illustrated in Figure 1, describes disc possession in Ultimate as a sequence of states characterized by field location. By including spatial representation of the playing field within the state transition model, we were able to properly apply the observational methodology and interpret the findings with consideration for game context (Barreira et al., 2020). The starting state in this model refers to any event that initiates a new possession, either a pull or an earned turnover. A turnover is earned when the opposing team commits a turnover, either forced or unforced, and play continues from where the turnover occurred. The playing field is divided into zones, which reflect the four discrete transient states (Figure 2); because the sample entities are taken from the AUDL, where games are often played on American football fields, the zones are defined using the yard lines: end zone (behind the 10-yard line), rear zone (between the 10- and 35-yard lines nearest the offensive team’s end zone), mid zone (between the two 35-yard lines), and front zone (between the 35- and 10-yard lines nearest the defensive team’s end zone). It is worth noting that the names of the transient states (zones) change depending on which team has possession of the disc, as the playing direction changes when disc possession changes (American Ultimate Disc League, 2019). The absorbing state is any event that terminates a possession, either a point or a turnover. In this context, all state transitions (except the pull transitions) occur every time the disc is passed.

**FIGURE 3:**
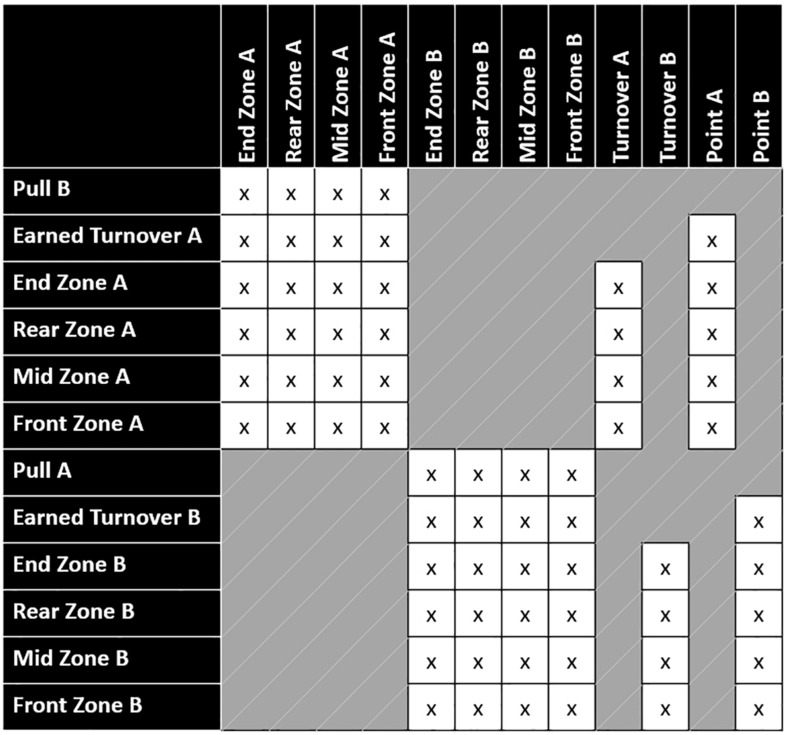
State transition matrix for all the possible transition probabilities (x) between the starting states, transient states, and absorbing states.

Transitions between states can occur within the same zone, between adjacent zones, and across zones; however, transitions are impossible between pulls and points, pulls and turnovers, and earned turnovers and turnovers (Figure 3). In rare instances, it is possible in Ultimate to score directly from a turnover (transition between earned turnover and point)—this is called a “Callahan” and occurs when an interception is caught inside the defender’s own end zone (American Ultimate Disc League, 2019). These transition probabilities are thus a function of the location on the field where the disc is held before being thrown. The turnover transitions represent where (i.e., in which zone) the turnover occurred. The earned turnover transitions indicate where a new possession begins. Similarly, the pull transitions refer to where the disc was caught or picked up by the receiving team.

### Reliability

Whereas some of the states are objective, for example, if a point was scored, others are more subjective, such as the exact position (zone) where a pass was caught or thrown and whether a turnover was forced or unforced. For this reason, a second experienced investigator annotated half of one game from our sample, consisting of 371 observations, and we were thus able to measure inter-rater reliability. Cohen’s kappa was calculated for all states, specifically for the classification of turnovers, and showed an agreement of 0.857 and 0.877. According to Landis and Koch (1977), these values demonstrate almost perfect inter-rater agreement.

### Data Analysis

Descriptive statistics were calculated for all nine performance indicators (PPG, BPG, CPG, PPP, TTPG, UTPG, FTPG, TTPCE%, and PO%) from the observational system. The state transition model annotations generated 30 different state transition probabilities. These state transitions can be classified as four pull probabilities (end, rear, mid, and front zones), four earned turnover probabilities (end, rear, mid, and front zones), six passing probabilities (end to rear, rear to mid, mid to front, front to mid, mid to rear, and rear to end), eight turnover probabilities (end, rear, mid, and front zones for forced and unforced turnovers), and four scoring probabilities (end, rear, mid, and front zones). The passing probabilities were also used to report the distribution of attempted passes thrown from each of the four zones (end, rear, mid, and front zones). As such, the state transition probabilities provide meaningful performance indicators, for example, the quality and tactical efficiency of pulls, passes, and points.

Descriptive statistics are provided as means and standard deviations, as well as medians and interquartile ranges, as some variables did not show a normal distribution according to a Shapiro–Wilk test. A comparison was made between winners (*n* = 14) and losers (*n* = 14). For the normally distributed variables, paired sample *t*-tests were used. For variables where the assumption of a normal distribution was violated, we used its nonparametric counterpart, the Wilcoxon signed-rank test for paired samples. Cohen’s d effect sizes were considered trivial (0–0.19), small (0.20–0.49), medium (0.50–0.79), and large (>0.80) (Cohen, 1992). Statistical significance was set at *p* < 0.05 (two-tailed). All statistical analyses were performed using SPSS (v27.0.1.0). The plots were created in R (v4.0.2), utilizing the following packages *via* RStudio (v1.3.1056): ggplot2 (v3.2.2), gggap (v1.0.1), and ggpattern (v0.1.3).

## Results

**FIGURE 4:**
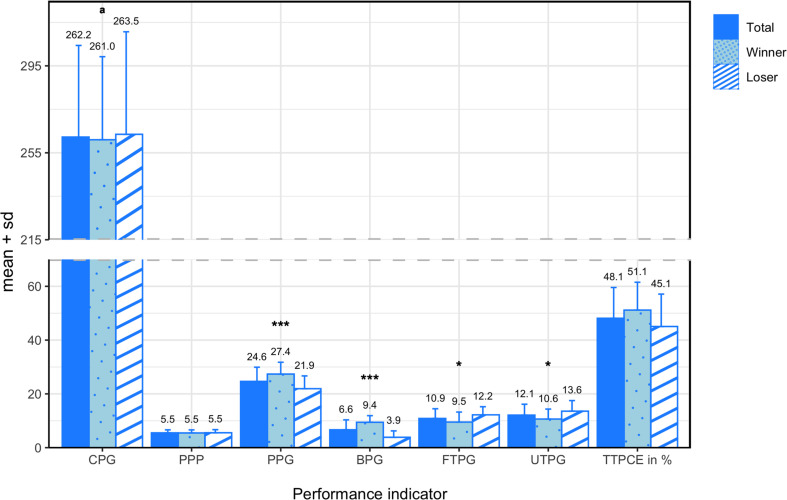
Means and standard deviations of all performance indicators for all teams, winning teams, and losing teams. * denotes a significant difference between winning and losing teams at a significance level of 0.05 and *** at a significance level of 0.001. Variables compared using a non-parametric test are marked with an ^*a*^.

Means and standard deviations of the nine performance indicators for all teams, winning teams, and losing teams are presented in Figure 4. Having an equal PO% of 50.00 ± 0.75%, teams scored an average of 24.64 ± 5.27 PPG. On average, teams recorded 262.25 ± 42.14 CPG and 5.50 ± 1.13 PPP. As for the average number of turnovers per game, teams committed 22.96 ± 5.49 TTPG, of which 12.11 ± 4.04 were UTPG and 10.86 ± 3.61 were FTPG. Teams converted an average of 48.11 ± 11.45% of their earned TTPCE%, resulting in 6.64 ± 3.70 BPG.

In the comparison between winning and losing teams, paired sample *t*-tests revealed significant differences in PPG (*M* = 5.43; *SD* = 3.65; *t*_(13)_ = 5.561; *p* < 0.001; *d* = 1.19), BPG (*M* = 5.57; *SD* = 3.28; *t*_(13)_ = 6.365; *p* < 0.001; *d* = 2.30), TTPG (*M* = −5.64; *SD* = 3.61; *t*_(13)_ = −5.852; *p* < 0.001; *d* = −1.18), UTPG (*M* = −2.93; *SD* = 4.65; *t*_(13)_ = −2.357; *p* = 0.035; *d* = −0.77), and FTPG (*M* = −2.71; *SD* = 3.95; *t*_(13)_ = −2.571; *p* = 0.023; *d* = −0.80). Although the paired sample *t*-test did not report a significant difference in TTPCE% (*M* = 6.09%; *SD* = 12.44%; *t*_(13)_ = 1.832; *p* = 0.090), a medium effect size was reported (*d* = 0.54). No significant difference was reported in PO% (*M* = 0.34%; *SD* = 1.49%; *t*_(13)_ = 0.858; *p* = 0.406), and only a small effect size was observed (*d* = 0.46). The Wilcoxon signed-rank test did not reveal a significant difference in CPG (Z = −0.251; *p* = 0.802), and the effect size was trivial (*d* = −0.06).

**FIGURE 5:**
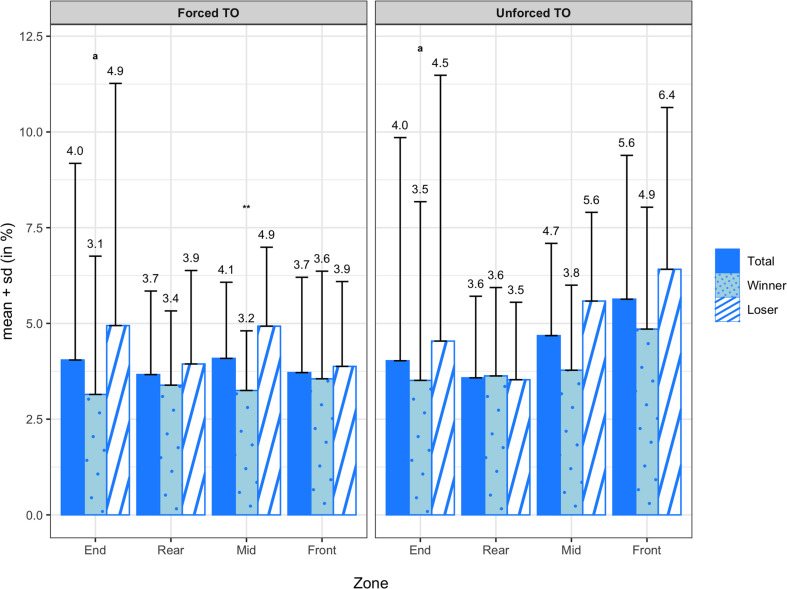
Transition probabilities of forced (4a) and unforced (4b) turnovers by zone for all teams, winning teams, and losing teams. ** denotes a significant difference between winning and losing teams at a significance level of 0.01. Variables compared using a non-parametric test are marked with an ^*a*^.

**FIGURE 6:**
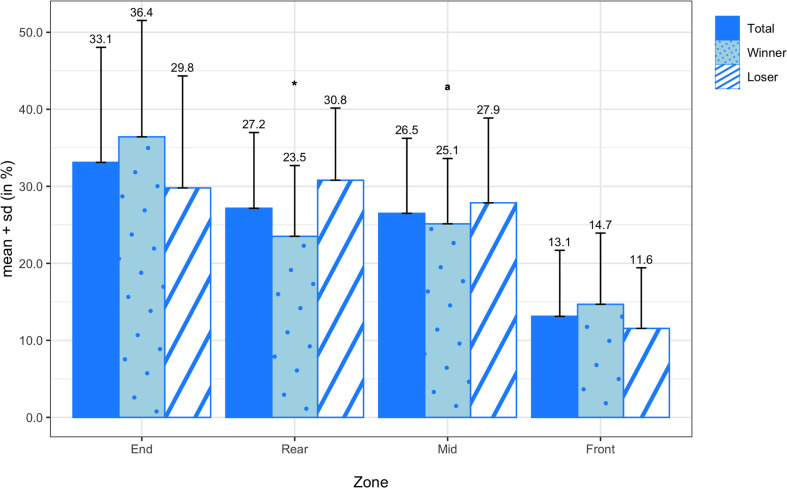
Transition probabilities of earned turnovers by zone for all teams, winning teams, and losing teams. * denotes a significant difference between winning and losing teams at a significance level of 0.05. Variables compared using a non-parametric test are marked with an ^*a*^.

Figure 5 summarizes the means and standard deviations of the forced and unforced turnover transition probabilities for all teams, winning teams, and losing teams. On average, forced turnovers were committed most frequently in the mid zone (4.09 ± 1.99%) and end zone (4.04 ± 5.14%), followed by the front zone (3.72 ± 2.49%) and rear zone (3.66 ± 2.18%). Unforced turnovers were committed most frequently in the front zone (5.63 ± 3.76%) and mid zone (4.68 ± 2.41%), followed by the end zone (4.02 ± 5.83%) and rear zone (3.58 ± 2.13%). When comparing winners and losers, the Wilcoxon signed-rank test reported a significant difference in forced turnovers in the mid zone (Z = −2.794; *p* = 0.005; *d* = −0.92). Although the paired sample *t*-test did not reveal a significant difference in unforced turnovers in the mid zone (*M* = −1.81%; *SD* = 3.30%; *t*_(13)_ = −2.045; *p* = 0.062), there was a medium effect size (*d* = −0.79). The means and standard deviations of the earned turnover transition probabilities are illustrated in Figure 6 for all teams, winning teams, and losing teams. On average, turnovers were earned most frequently in the end zone (33.11 ± 14.94%), followed by the rear zone (27.15 ± 9.83%) and mid zone (26.49 ± 9.75%) and finally the front zone (13.12 ± 8.57%). Between winning and losing teams, the paired sample *t*-test revealed a significant difference in earned turnovers in the rear zone (*M* = −7.29%; *SD* = 11.35%; *t*_(13)_ = −2.403; *p* = 0.032; *d* = −0.79).

**FIGURE 7:**
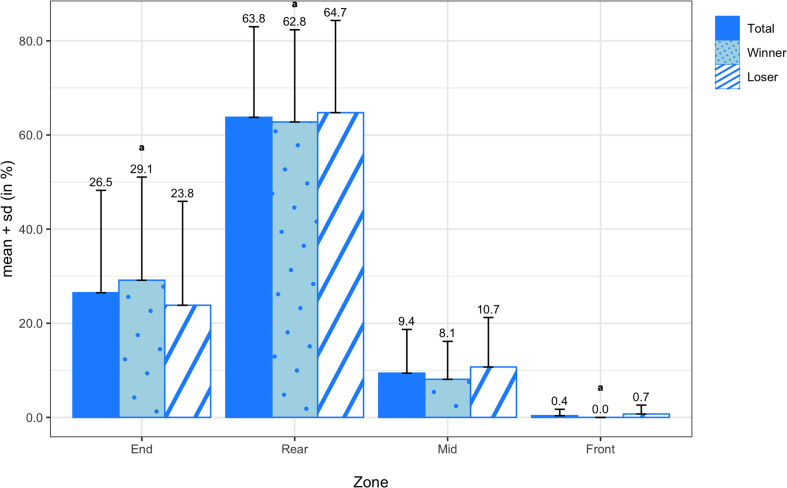
Transition probabilities of pulls by zone for all teams, winning teams, and losing teams. Variables compared using a non-parametric test are marked with an ^*a*^.

**FIGURE 8:**
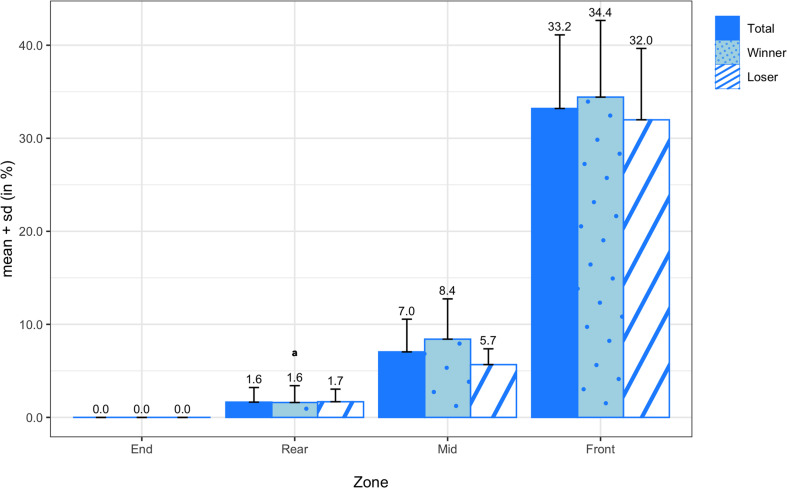
Transition probabilities of points scored by zone for all teams, winning teams, and losing teams. Variables compared using a non-parametric test are marked with an ^*a*^.

The means and standard deviations of the pull transition probabilities for all teams, winning teams, and losing teams are shown in Figure 7. On average, pulls were caught or landed most frequently in the rear zone (63.75 ± 19.25%), followed by the end zone (26.57 ± 21.71%), mid zone (9.39 ± 9.31%), and front zone (0.36 ± 1.34%). There were no significant differences in any of the pull probabilities between winners and losers, but there was a medium effect size in pulls in the end zone (*d* = 0.51). In Figure 8, the means and standard deviations of the scoring transition probabilities are reported for all teams, winning teams, and losing teams. On average, points were scored most frequently from the front zone (33.20 ± 7.91%), followed by the mid zone (7.04 ± 3.51%) and rear zone (1.64 ± 1.57%), and finally the end zone (0.00 ± 0.00%). Although the paired sample *t*-test did not reveal a significant difference in scoring from the mid zone (*M* = 2.74%; *SD* = 4.78%; *t*_(13)_ = 2.143; *p* = 0.052) between winning and losing teams, there was a large effect size (*d* = 0.83).

**FIGURE 9:**
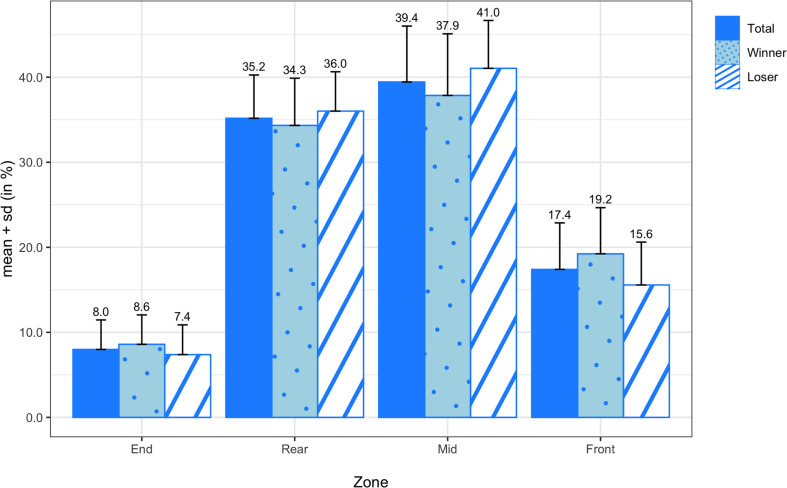
Transition probabilities of attempted passes by zone for all teams, winning teams, and losing teams.

**FIGURE 10:**
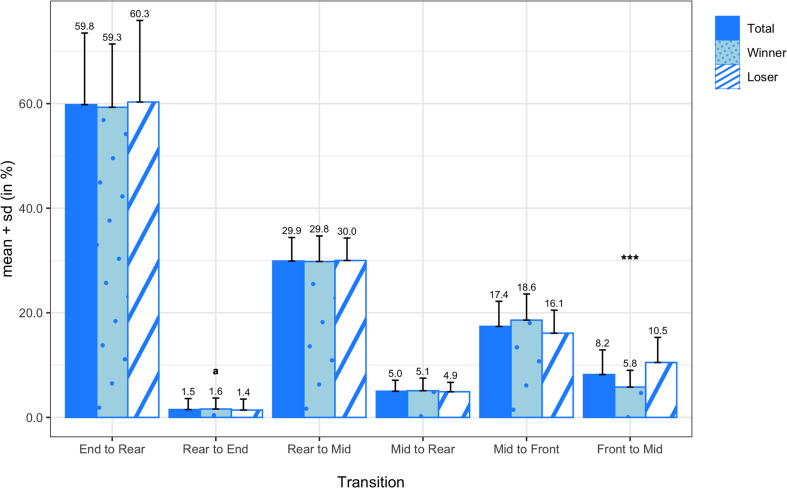
Transition probabilities of passing between adjacent zones for all teams, winning teams, and losing teams. *** denotes a significant difference between winning and losing teams at a significance level of 0.001. Variables compared using a non-parametric test are marked with an ^*a*^.

Figure 9 displays the means and standard deviations of the attempted passing probabilities for all teams, winning teams, and losing teams. On average, attempted passes were most frequently thrown from the mid zone (39.44 ± 6.57%) and rear zone (35.17 ± 5.09%), followed by the front zone (17.40 ± 5.47%) and end zone (7.99 ± 3.47%). There were no significant differences in any of the attempted passing probabilities between winners and losers, but there was a medium effect size in attempted passes from the front zone (*d* = 0.70). In Figure 10, the means and standard deviations of the passing probabilities between adjacent zones are depicted for all teams, winning teams, and losing teams. In the comparison between winning and losing teams, the paired sample *t*-test revealed a significant difference in backward passing from the front zone to the mid zone (*M* = −4.73%; *SD* = 3.55%; *t*_(13)_ = −4.980; *p* < 0.001; *d* = −1.16).

## Discussion

This is the first study on tactical performance analysis in Ultimate. Thus, the findings from this article only begin to reveal the unique nature of the sport and the variables that contribute to the strengths and weaknesses observed in elite Ultimate competition.

PO% was included in the analysis but not considered a true performance indicator in Ultimate, as we were able to confirm that the rules of Ultimate enable equal opportunity to disc possession for both teams. The mean difference in PO% between winners and losers was less than 0.4% and not statistically significant. Regarding the performance indicators, we found that PPG and BPG were significantly different between winners and losers, and large effects were observed for both. These indicators have been classified in previous work as general match indicators, which are those that provide basic information about performance (Hughes and Bartlett, 2002). This information is considered basic because the score is an inherent indicator of performance—for a team to win, it is a result of the game’s structure that more points must be scored. Breakpoints in Ultimate are akin to breaking serve in tennis, where a player is more likely to win their own service games, thus treating breakpoints (the point before breaking serve) as the most important points in the game (O’Donoghue and Liddle, 1998). In Ultimate, the team that receives the pull and starts on offense is essentially playing their own service game. So, when the team who throws the pull and starts on defense is the team to score, it could suggest that they are beating the odds and increasing their overall likelihood of winning the game. Similar to other game sports, the number of BPG cannot solely be attributed to the team who scores them, but also to their opponent, as their behavior influences the dynamic interactions that occur in Ultimate (Lames and McGarry, 2007).

Winners committed significantly fewer TTPG, UTPG, and FTPG than losers. In rugby union, a significant association between turnovers and winning/losing performance has already been reported (Bremner et al., 2013). This is a reasonable association, considering turnovers result in a loss of possession and scoring is only possible when a team is in possession. Given that all three of these technical indicators showed statistical significance, it can be said that winners in Ultimate were maintaining disc possession by both making fewer mistakes (UTPG) and by counteracting the defensive actions of their opponents (FTPG). In soccer, turnovers have been recognized as indicators of technical weaknesses (Hughes and Bartlett, 2002; Korte and Lames, 2018). In the present study, UTPG is defined as a loss of possession due to an error. The cause of these errors was not scrutinized in this study, but further analysis of the effectiveness of throwing and catching techniques in Ultimate could be conducted to determine their effect on unforced turnovers. From a psychological perspective, winners could be less susceptible to unforced errors due to characteristics such as mental toughness, as was reported in badminton by Yadav et al. (2007). For FTPG, it could also be the case that winners were not in fact counteracting their opponent’s defensive actions, but rather their opponents were simply making fewer attempts at provoking turnovers either due to lack of skill or poor choice of strategy. In any case, the results show that winners had significantly fewer forced turnovers than losers, and the probability of them occurring in the mid zone was statistically significant. Forced turnovers in Ultimate could thus benefit from not only a more in-depth technical analysis but also tactical, such as an analysis of the different types of defensive formations. Although not statistically significant, the state transition model also reported a large effect size in scoring from the mid zone and a medium effect size in unforced errors in the mid zone. These findings could suggest that the mid zone is where critical events in Ultimate occur, although it is worth noting that in the present state transition model, the mid zone is slightly larger than the other zones by 10 to 15 yards; the mid zone covers 30 yards, the rear and front zones cover 25 yards each, and the end zones cover 20 yards each.

As for passing behavior in Ultimate, the results from the state transition model show that winners had a significantly lower probability than losers when passing from the front zone to the mid zone. Passing is one of the most frequent actions in invasion sports, thus also making it one of the most important (Goes et al., 2019). Unlike rugby and American football, players in Ultimate can pass the disc in any direction—forward, backward, or laterally. However, players in Ultimate are not allowed to run with the disc. This unique combination of features from several sports, all played on similarly sized fields, creates an opportunity to understand the nuances that set Ultimate apart as its own invasion sport. Since passing backward is common in rugby, as passing forward is restricted, it would be more interesting to examine backward passing in soccer. Mindek et al. (2018) reported that backward passing occurred more frequently within the first-ranked teams in the English Premier League than the last-ranked teams during an eight-season period. The authors suggest that the first-ranked teams could be using backward passing as a method of maintaining ball possession; this reasoning is supported by the fact that soccer players are often dribbling the ball and must shield it from defenders. This is not applicable in Ultimate, as possession is secure as long as the player does not drop the disc. In this way, greater likelihood of backward passing from the front zone to the mid zone could be indicative of tactical weakness in losing teams, perhaps due to difficulty penetrating the defense. Conversely, less likelihood of backward passing near the opponent’s end zone could suggest stronger offensive plays by winning teams.

Reasonably, being the first look at performance analysis in Ultimate, there are certain limitations regarding this work. The first is that the present study focused on games played within the AUDL where the rules differ slightly from other associations (American Ultimate Disc League, 2019). Perhaps most notably, the sport of Ultimate is unique in that it is typically self-officiated (known as Spirit of the Game), but the AUDL uses referees to arbitrate game violations. Likewise, the sample entities were chosen due to accessibility. Thus, we did not control for potential confounding factors such as home advantage (Lago-Peñas and Lago-Ballesteros, 2011), as our sample included only five different home teams. However, the respective data already provide indications for potential home advantage in the AUDL, as home teams in our sample scored significantly more points (*M* = 5.00; *p* = 0.012) and committed significantly fewer turnovers (*M* = −5.18; *p* = 0.010).

Furthermore, the stability of the performance indicators must also be accounted for when the sport involves dynamic interactions between teams, such as Ultimate (Lames and McGarry, 2007). Although this interaction effect was considered in the interpretations of the performance indicators identified in this study, the reliability of these indicators has yet to be investigated. However, other work that has served as a starting point for such research in Ultimate included an evaluation of the validity and reliability of technical indicators used in the sport (Russomanno et al., 2016a,b), facilitating the future use of sport-specific terminology to ensure mutual understanding within performance analysis research. Future performance analysis studies in Ultimate should normalize the performance indicators, as suggested by Hughes and Bartlett (2002). For example, in this study, turnovers are normalized to the total number of games played, but it would also be of interest to normalize based on the total number of possessions, which could inform the ratio of possessions lost to the different types of turnovers. Finally, future research should also aim to increase the number of sample entities and to consider other Ultimate contexts (United States Ultimate, World Flying Disc Federation) to allow for large-scale investigations on the performance analysis of Ultimate. This could also enable researchers to apply promising approaches such as T-pattern analysis to identify re-occurring chronological patterns (Magnusson, 2020). This methodology, which was initially developed in the field of ethological and human interaction research, has already been applied to handball and boxing in the sport science literature (Pic, 2018; Pic and Jonsson, 2021). However, we understand the difficulty of obtaining such a large dataset due to there being only niche interest in the sport for now.

## Conclusion

This study is the first to apply state transition modeling that includes spatial information for tactical performance analysis outside of net games. By using states to represent different zones on the field, sports scientists will be better able to visualize disc movement within and between teams, facilitating our understanding of fundamental actions and events in Ultimate. Ideally, this approach will be used to examine tactical behaviors in other invasion sports. Future research can also build upon the findings of this work by expanding the sample to include elite-level Ultimate competition beyond the AUDL. This will hopefully further reveal the performance indicators that are uniquely relevant to Ultimate and can therefore contribute to the scientific knowledge base of this sport.

## Data Availability Statement

The original contributions presented in the study are included in the article/Supplementary Material, further inquiries can be directed to the corresponding author.

## Author Contributions

HL developed the observational system and the state transition model, collected the data, and performed a significant part of the statistical analysis. TR assisted in the development of the methodology and performed some statistical analyses. OK assisted in the development of the methodology and created the figures. ML assisted in the development of the methodology. All authors contributed to the manuscript.

## Conflict of Interest

The authors declare that the research was conducted in the absence of any commercial or financial relationships that could be construed as a potential conflict of interest.
